# Acute Anticholinergic Toxic Syndrome Due to Scopolamine Exposure: A Case Report with Possible Drug-Facilitated Administration

**DOI:** 10.3390/jox16030097

**Published:** 2026-05-29

**Authors:** Stanila Stoeva-Grigorova, Ivanesa Yarabanova, Maya Radeva-Ilieva, Galina Uzunova, Georgi Bonchev, Ivanka Gospodinova, Violeta Borisova, Elmira Vicheva, Simeon Marinov, Petko Marinov, Snezha Zlateva

**Affiliations:** 1 Department of Pharmacology, Toxicology and Pharmacotherapy, Faculty of Pharmacy, Medical University of Varna, 9000 Varna, Bulgaria; maya.radeva@mu-varna.bg (M.R.-I.); petko.marinov@mu-varna.bg (P.M.); snezha.zlateva@mu-varna.bg (S.Z.); 2 Clinical Toxicology Department, Naval Hospital, 9000 Varna, Bulgaria; ivanesa_98@abv.bg; 3 Laboratory of Analytical Toxicology, Naval Hospital, 9000 Varna, Bulgariageorgi.bontchev@gmail.com (G.B.);; 4 Department of Urology, Faculty of Medicine, Medical University of Varna, 9000 Varna, Bulgaria; dr.marinov.simeon95@gmail.com

**Keywords:** scopolamine, anticholinergic toxidrome, tropane alkaloids, GC–MS, intoxication, galantamine

## Abstract

Introduction: Scopolamine is a tropane alkaloid and non-selective muscarinic receptor antagonist capable of inducing a central anticholinergic toxidrome with pronounced neuropsychiatric and autonomic manifestations. Despite its well-characterized pharmacology, diagnosis remains clinically challenging due to variable presentation and often absent or unreliable exposure history, particularly in non-medical contexts. Methods: We report a case of a 34-year-old female presenting with an acute onset of altered mental status following a social event. Clinical findings included delirium, hallucinations, psychomotor agitation, dysarthria, bilateral mydriasis with preserved light reflex, sinus tachycardia, and marked urinary retention. Routine laboratory investigations were largely within normal limits, and standard urine toxicology screening was negative except for iatrogenic benzodiazepines. Given the characteristic toxidrome, extended toxicological analysis using gas chromatography–mass spectrometry (GC–MS) was performed, confirming the presence of scopolamine. Supportive treatment, including benzodiazepines and off-label intravenous galantamine, was administered, resulting in progressive clinical improvement and complete recovery without sequelae. Results: This case underscores the diagnostic limitations of routine toxicology screening for tropane alkaloids and highlights the importance of a pattern-based toxidrome approach. Given the absence of reliable exposure history and the presence of marked anterograde amnesia, an exogenous exposure cannot be excluded; however, no direct forensic evidence supports intentional administration. Furthermore, galantamine may represent a pharmacologically plausible alternative to physostigmine in settings where it is unavailable, although evidence remains limited. Conclusions: Scopolamine intoxication should be considered in cases of acute anticholinergic syndrome with negative standard screening, and confirmation requires advanced analytical methods such as GC–MS.

## 1. Introduction

Scopolamine is a tropane alkaloid naturally occurring in plants of the Solanaceae family, including *Atropa belladonna*, *Datura stramonium*, *Datura suaveolens*, *Hyoscyamus niger*, *Hyoscyamus muticus*, *Mandragora officinarum*, *Scopolia carniolica*, as well as *Duboisia myoporoides* and *Latua pubiflora* [1]. It is a non-selective competitive antagonist of muscarinic acetylcholine receptors (M1–M5), resulting in inhibition of parasympathetic neurotransmission and a predominance of anticholinergic effects. As a tertiary amine, it readily crosses the blood–brain barrier and exerts pronounced central nervous system activity [2,3]. At therapeutic doses, scopolamine may induce central nervous system depression, including sedation, amnesia, and suppression of vestibular function, whereas higher doses are associated with excitation, hallucinations, and irritability [2,4]. Peripheral manifestations include mydriasis, tachycardia, xerostomia, hyperthermia, urinary retention, and decreased gastrointestinal motility [3]. Severe intoxications may progress to central respiratory depression and life-threatening cardiac arrhythmias [5]. Scopolamine is clinically used for motion sickness and postoperative nausea and vomiting due to its vestibular inhibitory effects, with a maximum recommended oral daily dose of approximately 1.2 mg [6,7]. However, toxic exposure is more commonly encountered in non-medical settings, including ingestion of contaminated food products, fermented beverages, or plant material containing tropane alkaloids. Accidental exposure may occur due to morphological similarity between toxic and edible plants [8,9,10,11]. Reported oral doses associated with toxicity range from 0.5–3 mg for neuropsychiatric symptoms, while fatal outcomes have been described at 1–15 mg, with marked interindividual variability [2,12,13].

Beyond its medical use, scopolamine has historically been associated with central nervous system effects such as anterograde amnesia and hallucinations, contributing to its investigation as a psychoactive and experimental “truth serum” agent. In contemporary settings, plant-derived tropane alkaloids, particularly from the genus *Datura*, continue to be used recreationally despite significant toxicity risk [5,11]. Additionally, scopolamine has been reported as an adulterant in illicit psychoactive substances, increasing the risk of inadvertent exposure [14,15]. Since the 1950s, scopolamine (“burundanga”) has been described in cases of suspected drug-facilitated intoxication, including robbery and sexual assault. Its toxicodynamic profile—characterized by cognitive disorganization, hallucinations, and profound anterograde amnesia—results in increased vulnerability of exposed individuals [13,16,17,18]. Administration may occur via inhalation or addition to beverages, facilitated by its lack of distinctive taste or odor [17].

Although the central anticholinergic syndrome is a well-recognized toxicological entity, it remains diagnostically challenging due to heterogeneous clinical presentation, atypical manifestations, and frequently absent or unreliable exposure history [8,19]. This necessitates a high index of clinical suspicion, particularly in acute delirium with incongruent laboratory findings. In this context, the present report describes a case of acute anticholinergic toxidrome following scopolamine exposure in a non-medical setting, emphasizing diagnostic limitations and the importance of extended toxicological analysis in such presentations.

## 2. Case Presentation

### 2.1. Medical History

A 34-year-old female patient was admitted emergently due to an acutely developed disturbance of consciousness. According to accompanying individuals, following a social event, she briefly left the group and subsequently returned, complaining of marked fatigue. After a short sleep period (~1 h), she developed rapidly progressive neurological symptoms, including confusion, tremor, and hallucinations.

At the initial stage of history taking, no clear exposure to a toxic agent was identified. The patient denied the use of psychoactive substances; however, she did not exclude the possibility of inadvertent administration, which introduced substantial diagnostic uncertainty and raised suspicion of potential exposure to difficult-to-detect xenobiotics.

### 2.2. Physical Examination

Upon admission to the Emergency Department, the clinical presentation was consistent with an acute anticholinergic toxidrome. The patient was in a state of obnubilation, accompanied by marked psychomotor agitation. Behavioral disturbances were evident, including anxiety, fear, and inappropriate responses to external stimuli.

**Figure 1:**
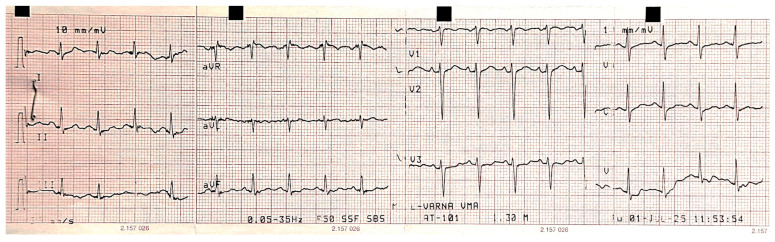
Patient’s electrocardiogram result on admission.

Neurological examination revealed dysarthria, confusion, and tremor. Bilateral mydriasis with preserved light reflexes was observed. Cardiovascular assessment demonstrated sinus tachycardia with a heart rate of 110–120 beats per minute and arterial blood pressure of 110/80 mmHg (Figure 1). Of particular note was pronounced urinary retention; approximately 2.5 L of urine were drained following catheterization, a finding strongly suggestive of a peripheral anticholinergic effect.

### 2.3. Paraclinical Investigations

Standard laboratory investigations were performed, including complete blood count, inflammatory markers, biochemical profile, and assessment of hepatic and renal function (Table 1). The complete blood count revealed leukocytosis (15.3 × 10^9^/L), while hemoglobin (Hb), platelet count, and erythrocyte indices remained within reference ranges, except for a slight decrease in erythrocyte count. C-reactive protein was within normal limits (1.04 mg/L), with no evidence of a significant systemic inflammatory response. The biochemical profile demonstrated mild hyperglycemia (6.63 mmol/L), with preserved renal function parameters (creatinine, urea) and liver enzymes aspartate aminotransferase (AST), alanine aminotransferase (ALT), and gamma-glutamyl transferase (GGT) within reference limits. Total bilirubin was at the upper limit of normal. Amylase levels were within the reference range.

Blood gas analysis was performed to evaluate acid–base status and gas exchange, including pH, partial pressures of oxygen and carbon dioxide (pO_2_, pCO_2_), bicarbonate concentration (HCO_3_^−^), base excess, oxygen saturation, and lactate levels (Table 2). All parameters were interpreted in relation to standard reference ranges and integrated into the overall toxicological assessment of the patient. Overall, the laboratory findings did not indicate organ dysfunction, with isolated leukocytosis representing the main abnormality in the context of an acute toxicological clinical presentation. The observed leukocytosis and mild hyperglycemia were interpreted as stress-related physiological responses in the context of acute anticholinergic intoxication and psychomotor agitation, rather than evidence of infectious or metabolic pathology, particularly in the absence of elevated inflammatory markers or organ dysfunction.

The urine sample was collected approximately 10–11 h after the onset of symptoms, corresponding to the acute post-exposure phase relevant for toxicological confirmation. The standard 10-panel urine toxicological screening was negative for most of the tested substances, with the exception of benzodiazepines, which is fully consistent with the iatrogenic sedation administered after hospital admission (Table 3). Extended immunoassay testing for additional psychoactive substances, including fentanyl, ketamine, phencyclidine, pregabalin, and tricyclic antidepressants, also yielded negative results.

Subsequently, gas chromatography–mass spectrometry (GC–MS) analysis of urine was performed. For the identification of xenobiotics, a 4 mL urine sample was processed using a routine GC–MS toxicological screening procedure. The workflow included initial deproteinization with 500 µL acetonitrile, followed by liquid–liquid extraction with 4 mL ethyl acetate under alkaline conditions. After phase separation, the organic layer was collected, dried over 100 mg anhydrous MgSO_4_, and centrifuged for 2 min at 4000 rpm. The solvent was evaporated to dryness under a nitrogen stream at 60 °C, and the residue was reconstituted in 50 µL methanol. GC–MS injection volume was 1 µL. Instrumental conditions are summarized in Table 4.

**Figure 2:**
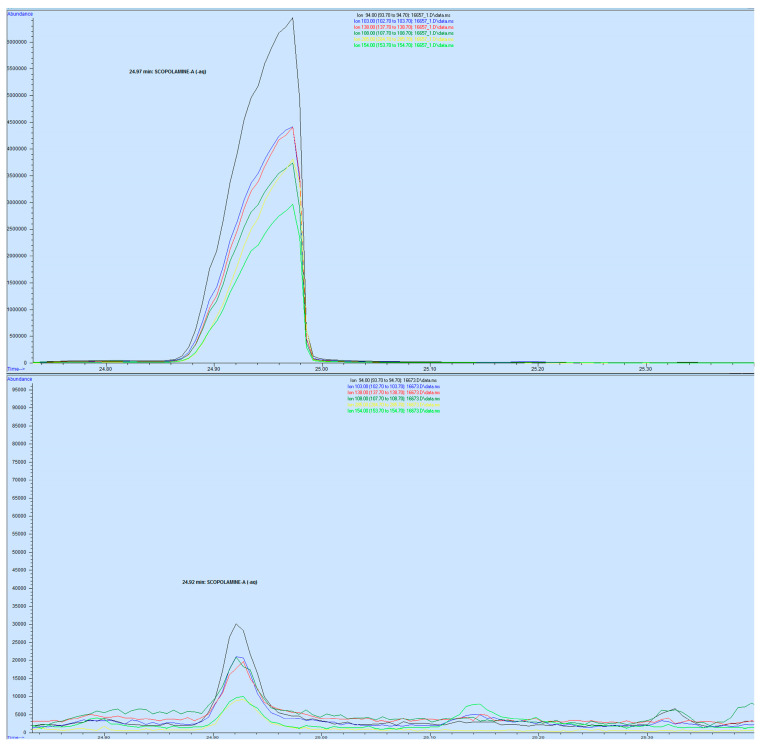
Comparison of the GC-MS extracted ion chromatograms of two urine samples, taken on the day of hospital admission (**up**) and the next day (**below**).

GC–MS analysis revealed the presence of scopolamine and lidocaine in the urine sample collected at admission. Lidocaine was interpreted as iatrogenic, related to urinary catheterization. Scopolamine identification was performed using full-scan GC–MS analysis following the described extraction procedure, and was based on retention time matching, characteristic electron ionization fragmentation pattern, and spectral concordance with the NIST and Wiley mass spectral libraries. The identification was considered reliable when all chromatographic and spectral criteria were fulfilled. The method was applied as a qualitative screening approach rather than a quantitative assay. Follow-up serial urine analyses demonstrated a progressive decrease in scopolamine signal intensity, with a detectable but non-quantifiable level on day 2 and complete disappearance by day 3 (Table 5, Figure 2).

### 2.4. Clinical Course

The clinical course was characterized by dynamic neurological manifestations, including retrograde amnesia involving approximately 20 h, persistent mydriasis, blurred vision, dysarthria, tremor, and episodes of psychomotor agitation. Despite initial sedation, symptoms persisted, suggesting ongoing central anticholinergic activity. Following pharmacological sedation, a transient sleep period of approximately 2.5 h was observed. Upon awakening, partial clinical improvement was noted; however, significant confusion persisted, along with partial retrograde amnesia, with the patient unable to recall events from the preceding ~20 h.

### 2.5. Diagnosis

Based on the clinical presentation at admission, characterized by both central and peripheral anticholinergic manifestations—including confusion, psychomotor agitation, hallucinations, mydriasis with preserved light reflexes, sinus tachycardia, and marked urinary retention—a working diagnosis of acute anticholinergic toxidrome was established.

The diagnostic process was complicated by the absence of a clear exposure history and initially negative standard toxicological screening results. Nevertheless, given the typical constellation of central and peripheral signs, extended toxicological investigations were performed to identify the causative agent. Ultimately, a definitive diagnosis of acute anticholinergic toxidrome was established, most likely secondary to scopolamine exposure.

### 2.6. Treatment

The therapeutic approach was complex and included both symptomatic and antidotal treatment (Table 6). Benzodiazepines were administered for control of psychomotor agitation, and haloperidol was given intramuscularly. Supportive therapy included intravenous fluid administration, vitamin B complex supplementation, nootropic support, and parenteral nutrition.

Following confirmation of scopolamine exposure, antidotal therapy with galantamine was initiated at a total dose of 30 mg intravenously. As a centrally acting acetylcholinesterase inhibitor, galantamine restores cholinergic neurotransmission and functionally antagonizes the anticholinergic effects of scopolamine. In the present case, clinical improvement was observed following administration; however, this does not allow definitive conclusions regarding therapeutic efficacy.

### 2.7. Outcome

The patient was discharged in a stable haemodynamic and afebrile condition, without residual signs of toxic syndrome. At the time of discharge, cognitive function was normalized, with full restoration of orientation and resolution of psychomotor agitation. No further delirious or psychotic symptoms were observed. Vital parameters remained within physiological limits, and neurological examination was unremarkable.

A favorable clinical evolution was noted under supportive and symptomatic therapy, with complete regression of anticholinergic manifestations. The patient was discharged with recommendations to avoid exposure to alcohol and psychoactive substances. Scheduled follow-up examinations were arranged for clinical monitoring.

## 3. Discussion

The presented case demonstrates an acute anticholinergic toxidrome characterized by prominent central and peripheral manifestations, including delirium, hallucinations, mydriasis, tachycardia, and urinary retention. The clinical picture was accompanied by a negative standard toxicological screening, necessitating extended analytical verification. This clinical entity is typical of exposure to tropane alkaloids, in which symptom severity often does not correlate with routine laboratory abnormalities [5,19]. The main differential diagnostic considerations included:

○ anticholinergic toxidrome (drug- or plant-induced),
○ delirium secondary to infectious processes,
○ acute neurological disorders (e.g., encephalitis, status epilepticus),
○ intoxication with other psychoactive substances.

The absence of inflammatory markers, together with a normal acid–base status and the presence of a characteristic combination of central (delirium, hallucinations) and peripheral (mydriasis, tachycardia, urinary retention) anticholinergic manifestations, strongly supports a toxicological rather than infectious or metabolic etiology. In this context, the diagnosis is fundamentally pattern-based, relying on recognition of muscarinic receptor antagonism rather than laboratory confirmation, which remains largely non-specific and primarily exclusionary [20].

Standard toxicological screening does not include tropane alkaloids, which represents a significant diagnostic limitation. This creates a substantial risk of false-negative results and diagnostic delay [19]. Therefore, the diagnostic approach in such patients should be based on three key pillars: (1) recognition of the toxidrome, (2) assessment of clinical probability, and (3) early application of extended analytical methods. Scopolamine identification in clinical and forensic toxicology is most robustly achieved by chromatographic–mass spectrometric techniques, with liquid chromatography–tandem mass spectrometry (LC–MS/MS) generally considered the reference method due to its superior sensitivity and selectivity for polar and thermolabile compounds. GC–MS, when combined with appropriate derivatization or artifact formation during sample preparation, may also provide reliable qualitative confirmation, particularly in retrospective screening workflows. Therefore, the combined use of LC–MS/MS and GC–MS approaches is complementary rather than mutually exclusive, depending on laboratory infrastructure and analytical objectives [2,4]. Although scopolamine is characterized by a short plasma elimination half-life (approximately 1–4 h), several forensic and clinical case reports have demonstrated that urinary detection may persist significantly beyond the expected pharmacokinetic window. Positive urine findings have been reported up to 18–48 h post-exposure, even in cases where plasma concentrations were already negligible or undetectable [21,22,23]. In addition, they enable retrospective toxicological analysis (e.g., hair samples) in cases where exposure cannot be documented at the time of presentation [9,13]. In the present case, GC–MS was decisive, confirming exposure to a compound outside routine screening panels and resolving diagnostic uncertainty. However, from a clinical and forensic perspective, toxicokinetic analysis alone does not allow a reliable differentiation between accidental exposure (e.g., ingestion of plant-derived scopolamine) and deliberate administration in drug-facilitated crimes. While pharmacokinetic data may assist in interpreting exposure timing and elimination patterns, they lack specificity regarding the route and intent of exposure. Therefore, such differentiation requires an integrated evaluation of toxicological findings, clinical presentation, temporal consistency of symptoms, and contextual investigative information.

From a therapeutic perspective, the management of anticholinergic toxidrome is based on three fundamental principles: supportive care, control of central agitation, and specific antidotal therapy. Supportive measures include airway stabilization, management of hyperthermia, prevention of rhabdomyolysis, and continuous cardiac monitoring. Physostigmine, the standard antidote for central anticholinergic syndrome, was not available in the country; therefore, galantamine was used as an alternative centrally acting acetylcholinesterase inhibitor [24,25,26,27]. Galantamine is a tertiary alkaloid that readily penetrates the central nervous system and exerts dual pharmacological activity. In addition to reversible acetylcholinesterase inhibition, it acts as an allosteric potentiating ligand of neuronal nicotinic acetylcholine receptors, thereby enhancing cholinergic neurotransmission and potentially counteracting central manifestations of muscarinic receptor blockade. Compared with physostigmine, galantamine has a longer elimination half-life and a slower pharmacodynamic profile, which may result in more sustained central cholinergic modulation but a delayed onset of clinical effect [28,29,30,31,32]. In contrast to physostigmine, which typically induces clinical improvement within minutes, galantamine may require several hours before observable symptom reversal, as demonstrated in the present case. However, although galantamine is not considered a standard antidote for anticholinergic syndrome, its potential use in anticholinergic intoxication is officially recognized in the Summary of Product Characteristics published by the Bulgarian Drug Agency, where its applicability in anticholinergic drug poisoning is specifically stated under appropriate clinical conditions [33]. Furthermore, despite pronounced central anticholinergic manifestations, the patient remained hemodynamically stable and did not require aggressive airway management or intensive care-level sedation, allowing consideration of a centrally acting acetylcholinesterase inhibitor as part of a targeted therapeutic approach. Supportive therapy alone was considered potentially insufficient due to the persistence of neuropsychiatric manifestations, including prolonged confusion, hallucinations, dysarthria, and psychomotor agitation. The choice of galantamine was additionally supported by regional clinical experience with the compound and its documented pharmacological rationale in anticholinergic intoxication [6,34,35]. Although a direct causal relationship between galantamine administration and clinical recovery cannot be definitively established in a single case report, the observed temporal association supports the pharmacological plausibility of galantamine as an alternative antidotal approach when physostigmine is unavailable.

From an etiological perspective, the absence of available pharmaceutical formulations of scopolamine in Bulgaria and the lack of evidence for iatrogenic exposure suggest an exogenous non-pharmaceutical source, including possible accidental or intentional administration. However, interpretation is limited by the frequent inability of patients to provide a reliable exposure history due to anterograde amnesia and altered mental status, requiring repeated and structured anamnesis [19]. Given the characteristics of the present case, drug-facilitated exposure may be considered as a theoretical possibility; however, it cannot be confirmed based on the available clinical and toxicological data. An alternative source may include plant-derived tropane alkaloids (e.g., *Datura*, *Hyoscyamus*, *Atropa*) through ingestion of herbal preparations or contaminated food; however, this is less likely, as such exposure would typically result in detection of multiple alkaloids in biological samples [5,36,37].

Taken together, these cases highlight the necessity of a multidisciplinary approach involving emergency medicine, clinical toxicology, and forensic toxicology to ensure reliable diagnostic confirmation and medicolegal assessment [13,17,38,39]. Despite the limitation of missing quantitative toxicokinetic data, the robust clinico-analytical concordance provided by GC–MS remains sufficient to substantiate the diagnosis.

## 4. Conclusions

The presented case illustrates a typical yet diagnostically challenging anticholinergic toxidrome induced by scopolamine, in which the severity of the clinical manifestations significantly exceeded the abnormalities detected in standard laboratory parameters. This underscores the limited diagnostic utility of routine toxicological screening and the need for a clinically driven diagnostic approach. A key element in establishing the diagnosis was the application of high-specificity GC–MS, which confirmed exposure to a substance not included in standard toxicological panels. This finding highlights the importance of extended analytical diagnostics in suspected exposure to tropane alkaloids.

The rapid clinical response observed following administration of a centrally acting acetylcholinesterase inhibitor supports the functional, receptor-mediated nature of the intoxication. In this context, galantamine may represent a potential therapeutic alternative in settings where physostigmine is unavailable, although its use should be considered off-label, based on pharmacodynamic rationale and observed clinical effectiveness.

From a forensic perspective, scopolamine intoxication should also be considered a potential marker of exposure in non-medical contexts, including accidental or intentional administration, particularly in patients presenting with pronounced anterograde amnesia and unreliable history. This necessitates a multidisciplinary approach, including evaluation for possible non-therapeutic exposure.

In conclusion, scopolamine should be included in the differential diagnosis of acute anticholinergic syndrome with a negative standard toxicological screen. Early clinical recognition and the use of confirmatory analytical methods are essential for accurate diagnosis and appropriate therapeutic management.

## Figures and Tables

**Table 1:** Hematological and biochemical parameters.

Parameter	Value	Reference Range
Hb [g/L]	136	130–180
Erythrocytes [×10^12^/L]	4.38	4.8–6.2
Hematocrit [L/L]	0.399	0.35–0.55
Leukocytes [×10^9^/L]	15.3	3.5–10.5
Platelets [×10^9^/L]	369	140–440
MCV [fL]	91.1	82–100
MCH [pg]	31.1	28–32
MCHC [g/L]	341	300–360
RDW [%]	13.8	11.5–14.9
CRP [mg/L]	1.04	<5
Glucose [mmol/L]	6.63	4.1–5.9
Creatinine [µmol/L]	69	64–104
Urea [mmol/L]	3.07	2.8–7.2
Total bilirubin [µmol/L]	20.25	5–21
AST [U/L]	21.81	<35
ALT [U/L]	10.92	<50
GGT [U/L]	7.62	<55
Amylase [U/L]	57.45	28–100

Hb—hemoglobin; MCV—mean corpuscular volume; MCH—mean corpuscular hemoglobin; MCHC—mean corpuscular hemoglobin concentration; RDW—red cell distribution width; CRP—C-reactive protein; AST—aspartate aminotransferase; ALT—alanine aminotransferase; GGT—gamma-glutamyl transferase.

**Table 2:** Blood gas analysis and acid–base status.

Parameter	Value	Reference Range
pH	7.45	7.35–7.45
pCO_2_ [kPa]	4.1	4.7–6.0
HCO_3_^−^ (actual) [mmol/L]	20.8	22–26
Base excess (ECF) [mmol/L]	−2.0	±3
pO_2_ [kPa]	10.7	10–13
O_2_ saturation [%]	96.4	95–100
Lactate [mmol/L]	0.5	0.5–2.2

pH—potential of hydrogen; pCO_2_—partial pressure of carbon dioxide; pO_2_—partial pressure of oxygen; HCO_3_^−^—bicarbonate; ECF—extracellular fluid; O_2_—oxygen.

**Table 3:** Results from the 10-panel urine drug screening test (Hangzhou Alltest Biotech Co., Ltd., Hangzhou, Zhejiang Province, China).

Substance	Cut-Off (Urine, ng/mL)	Result
Amphetamine	1000	−
Cocaine (Benzoylecgonine)	300	−
Δ9-Tetrahydrocannabinol metabolite (Marijuana)	50	−
Benzodiazepines	300	+
Tricyclic Antidepressants	1000	−
Barbiturates	300	−
Morphine (Opiates)	300	−
Methadone	300	−
Methamphetamine	1000	−
3,4-Methylenedioxymethamphetamine	500	−
Fentanyl	20	−

*“+” indicates positive result above cut-off concentration; “−” indicates negative result below cut-off threshold; Cut-off values represent immunoassay detection limits provided by the manufacturer.*

**Table 4:** GC-MS Operation conditions, SCAN mode.

Parameter	Value	Parameter	Value
Initial oven temp.	50 °C	GC Column	DB-1701
Initial time	2 min	Column dimensions	30 m × 0.250 mm
Oven ramp rate	20 °C min^−1^	Film thickness	0.25 μm
Oven final first ramp	90 °C	Inlet mode	splitless
Final time first ramp	1 min	Flow mode	constant flow
Oven ramp rate	8 °C min^−1^	Flow rate	1.5 mL min^−1^
Oven final temp.	280 °C	Carrier gas	No
Final time	15 min	Ion source temp.	230 °C
Total run time	43.75 min	Inlet temp.	250 °C

**Table 5:** Extended toxicological analysis (GC–MS).

Day	Result
01	Scopolamine (+), lidocaine (+)
02	Scopolamine (trace)
03	Scopolamine (−)

*“+” indicates detectable presence of compound above analytical threshold; “trace” indicates detectable but non-quantifiable concentration; “−” indicates absence below detection limit; Lidocaine detection was interpreted as iatrogenic, related to urinary catheterization procedure.*

**Table 6:** Therapeutic interventions.

Day	Therapeutic Goal	Treatment (Dose and Route)
01	IV hydration and electrolyte balance	○ Sodium chloride 0.9% (500 mL i.v.) ○ Glucose 5% (500 mL i.v., 4×/day) ○ Famotidine (i.v., 2×/day)
	Neurometabolic therapy	○ Piracetam (3 g i.v., 2×/day) ○ Vitamin B1 (thiamine) (i.v., 3×/day) ○ Vitamin B6 (pyridoxine) (i.v., 3×/day)
	Sedation/control of psychomotor agitation	○ Diazepam (1 ampoule i.m., as needed) ○ Haloperidol (1 ampoule i.m., as needed)
	Parenteral nutrition	○ Kabiven (24-h infusion)
02	Hydration and electrolyte balance	○ Sodium chloride 0.9% (500 mL i.v., 2×/day) ○ Glucose 5% (500 mL i.v., 2×/day) ○ Ringer’s solution (500 mL i.v., 1×/day)
	Acetylcholinesterase inhibitor therapy	○ Galantamine (Nivalin^®^, 10 mg/mL Sopharma AD, Sofia, Bulgaria): total 30 mg administered intravenously in a stepwise regimen (½ ampoule + ½ ampoule + 1 ampoule + 1 ampoule; i.e., 5 mg + 5 mg + 10 mg + 10 mg)
	Neurometabolic support	○ Vitamins B1 and B6 (i.v., 2×/day)
03	Clinical monitoring and stabilization	○ No active pharmacotherapy
		○ Supportive care

## Data Availability

The original contributions presented in this study are included in the article. Further inquiries can be directed to the corresponding author.

## Abbreviations

The following abbreviations are used in this manuscript:

ALT	Alanine aminotransferase
AST	Aspartate aminotransferase
CRP	C-reactive protein
ECG	Electrocardiogram
ECF	Extracellular fluid
GC–MS	Gas chromatography–mass spectrometry
GGT	Gamma-glutamyl transferase
Hb	Hemoglobin
HCO_3_^−^	Bicarbonate
LC–MS/MS	Liquid chromatography–tandem mass spectrometry
MCH	Mean corpuscular hemoglobin
MCHC	Mean corpuscular hemoglobin concentration
MCV	Mean corpuscular volume
O_2_	Oxygen
pCO_2_	Partial pressure of carbon dioxide
pO_2_	Partial pressure of oxygen
RDW	Red cell distribution width
